# Albuminuria - marker of progressive renal disease

**Published:** 2012-12-25

**Authors:** M Stoian, N State, V Stoica, G Radulian

**Affiliations:** * “Carol Davila" University of Medicine and Pharmacy, Bucharest, Internal Medicine Department, "Dr. Ioan Cantacuzino" Clinical Hospital, Bucharest; ** “Carol Davila" University of Medicine and Pharmacy, Bucharest, "N. C. Paulescu" Institute of Diabetes, Nutrition and Metabolic Diseases, Bucharest

**Keywords:** albuminuria, progressive renal disease, PI 3-kinase, pp70 kinase

## Abstract

The presence of albuminuria has long been recognized as an adverse prognostic feature in patients with renal disease: the patients with appreciable albuminuria are much more likely to develop tubulointerstitial scarring and fibrosis and progress to end-stage renal failure. For many years, it was thought that excess albuminuria was simply a marker of a more severe renal disease, which was more likely to progress as a result of this severity rather than as a result of the albuminuria itself. This conviction was strengthened by the general assumption that albumin was a benign or inert molecule serving primarily to exert oncotic pressure and act as a carrier within the circulation. More recently, this view has been challenged with the accumulation of evidence suggesting that albumin is able to influence the function of cells with which it makes contact in the manner of a signalling molecule.

Abbreviations

PTC =Proximal Tubular Epithelial Cells; PDZ acronym combining the first letters of three proteins =post synaptic density protein (PSD95), Drosophila disc large tumor suppressor (Dlg1), and zonula occludens-1 protein (zo-1); DNA=Dexoxyribonucleic Acid; PI 3-kinase= Phosphatidylinositol 3-kinase; mRNA= messenger Ribonucleic Acid.

## Proximal tubular cell binding and uptake of albumin

A number of morphological studies have established that albumin is reabsorbed by proximal tubular epithelial cells (PTC) by receptor-mediated endocytosis [**1**]. The majority of this reabsorbed albumin is subject to lysosomal degradation and the released amino acids reabsorbed into the circulation [**2**].

 The identity of the albumin receptors in PTC is not yet fully resolved. Compelling evidence indicates that megalin mediates at least a portion of albumin binding in PTC [**3**], although binding studies suggest the presence of at Ieast two binding sides for albumin in these cells [**4,5**]. Ligand blotting experiments indicate that a number of lower-molecular weight proteins may also contribute to albumin binding activity in the proximal tubule [**5**].

Megalin, a single polypeptide of 4660 amino acids, is a member of the low-density lipoprotein receptor family, and as such, its extracellular domain possesses many similarities with the other members of this family [**6**]. The accepted function of these receptors is intersperse of a variety of diverse ligands prior to lysosomal breakdown. Thus, megalin is a candidate for albumin binding in the proximal tubule. Interestingly, the cytoplasmic tail of megalin has no sequence homology with other members of the low-density lipoprotein receptor family except for short internalization sequences. This portion of the molecule, however, does contain Src-homology domains, PDZ domains, and a number of protein kinase phosphorylation sites [**6**]. Sequences such as these, strongly indicate a potentially novel role for megalin in signal transduction. Whether albumin binding to megalin transduces signals to the cell interior is unclear but is currently under investigation.

##  Non-kidney cell responses to albumin exposure

 Rupture of the blood-brain barrier exposes cells of the nervous system to unusually high concentrations of albumin, and is thus analogous to the situation faced by PTC in nephrosis. Astrocytes exposed to albumin display a number of responses, and it has been postulated that these albumin-evoked responses may contribute to the development of cerebral scarring after brain haemorrhage. Exposure of astrocytes to lipid-free albumin precipitates a marked and sustained reduction of intracellular calcium. The mechanism appears to be via an as yet unidentified albumin receptor stimulating an intracellular messenger to cause calcium to enter intracellular stores [**7**]. If serum albumin, completed with bound fatty lipids, is applied to astrocytes, calcium spiking is observed together with an increase in DNA synthesis [**7,8**].

In the circulation, interaction of albumin with endothelial cells critically regulates vascular permeability by modulating intracellular calcium concentrations [**9**]. Furthermore, binding of albumin to the endothelium activates an intracellular tyrosine kinase cascade [**10**]. This protein tyrosine kinase pathway probably regulates transcytosis of albumin across the endothelial monolayer but it is also likely to have other yet unidentified effects on cell function. One such effect may relate to cell survival. If cultured endothelial cells are starved of serum, they rapidly undergo apoptosis.

Incubation with albumin abrogates this apoptotic response in a specific manner at physiologically relevant concentrations [**11**]. These authors postulated that the albumin's ability to act as an endothelial survival factor was not due to its absorptive properties, but was rather a receptor-mediated event.

## Proximal tubular cell responses to albumin exposure in vitro

Several years ago, it was demonstrated that albumin was able to stimulate proliferation of cultured PTC [**12**]. Clues to the mechanism of this mitogenic effect were obtained from studies into the regulation of albumin endocytosis by Opossum kidney PTC (OK cells). It was demonstrated that receptor-mediated endocytosis of albumin by these cells was not only regulated by the p85/p110 phosphatidyl-inositol 3-kinase (PI 3-kinase), but that incubation or OK cells with albumin also stimulated PI 3-kinase activity [**13**]. Subsequently, together with the use ultrapure yeast recombinant human albumin, it was demonstrated that albumin possessed the ability to stimulate a kinase cascade involving PI 3-kinase upstream of pp70s6 kinase [**14**]. Furthermore, incubation of OK cells with albumin results in activation of p44/p42 extracellular signal regulated mitogen activated protein kinase [**15**], the activity of all these enzymes being essential for albumin-evoked mitogenesis in OK cells.

 Appreciable stimulation of PI 3-kinase and pp70s6 kinase was observed with incubated albumin concentrations as low as 1-10 μg/ml [**14**]. In health, albumin is not totally excluded from glomerular filtrate, and accordingly micropuncture studies have revealed albumin concentrations in the range of 10-30 μg/ml in the proximal tubules of rodents. Taken together, these intriguing results suggest that low concentrations of albumin in tubular fluid may have an important role in the maintenance of PTC integrity and homeostasis in health. Conversely, chronically increased albumin stimulation in nephrosis may disturb this homeostatic balance, thus favouring the development of the typical renal histological changes of progressive tubulointerstitial disease.

 Other studies have demonstrated a survival factor like the effect of albumin both in macrophages and mouse primary cultures of PTC. This was not associated with activation of PI 3-kinase, but appeared to be due to the ability of albumin to scavenge oxygen-free radicals accumulating in serum-free culture conditions [**16**]. Very recently, preliminary data has been presented indicating that albumin treatment of human immortalized PTC results in increased levels of hydrogen peroxide [**17**]. Clearly, some tension exists between the results of these various studies. Although differences in the models used may explain the discrepancies. More work is required to clarify this important area.

 As well as the stimulation of intracellular kinase cascades, it is clear that exposure to albumin leads to phenotypic changes in PTC. In vitro studies have shown that a number of pro-inflammatory and vasoactive genes are upregulated in PTC exposed to albumin. Overload of cultured PTC with albumin provokes synthesis and release of endothelin-1 into the basolateral compartment [**18**]. Among the chemoattractants both monocyte chemoattractant protein-1 and RANTES are released by rat PTC in response to albumin exposure [**19,20**]. The production of these chemokines by PTC is dependent on albumin-induced activation of the transcription factor family nf-β [**19,20**]. Indeed, substantial evidence has established that the NF-β family of proteins controls the expression of many genes regulating immunological, inflammatory and growth related cellular responses [**21**]. It seems likely therefore, that albumin induction of NF-β in PTC may lead to the expression of a range of genes relevant to the development of tubulointerstitial inflammation and scarring.

## Proximal tubular responses to albumin in vivo

The effect of proteinuria on PTC was studied in the non-immunologically mediated protein overload nephrosis model in rats [**22**]. Daily injection of 2 g of bovine serum albumin into the peritoneal cavity of rats renders the animals heavily proteinuric such that after 7 days they excrete up to 1 g urinary protein in 24 h. If sacrificed at this stage, considerable evidence of PTC proliferation is observed in proteinuric animals compared to controls, as assessed by in situ hybridization for histone mRNA [**22**]. This observation is in accordance with the effects of albumin on PTC proliferation seen in vitro. Predictably, the situation in the in vivo proximal tubule is more complex than that observed in cell culture. Marked apoptosis accompanies the proliferation seen in vivo in protein-overload nephrosis [**22**]. The stimulus for PTC-programmed cell death in proteinuria is unclear, but the contribution of albumin-bound fatty acids is likely to be important.

 Interstitial inflammation is observed in protein-overload nephrosis and is represented by a conspicuous macrophage infiltrate [**23**]. By careful immunohistochemical analysis of kidneys from proteinuric rats, Abbate et al. [**22**] showed that, regardless the type of glomerular injury, interstitial macrophages are found preferentially in association with those proximal tubules displaying the most marked accumulation of intracellular protein. Furthermore, co-localization of the chemokine osteopontin with macrophage infiltrates and protein-overloaded tubules was observed [**23**].

##  Summary

 A considerable body of evidence now implicates albumin and other filtered proteins in the pathogenesis of tubulointerstitial disease in the glomerulonephrites. Manipulation of the cell signalling and proinflammatory effects of albumin on PTC may prove an effective approach to therapy of progressive renal disease.
